# No Differences in Levels of Circulating Progenitor Endothelial Cells or Circulating Endothelial Cells Among Patients Treated With Ticagrelor Compared With Clopidogrel During Non–ST‐Segment–Elevation Myocardial Infarction

**DOI:** 10.1161/JAHA.118.009444

**Published:** 2018-09-20

**Authors:** Alejandro Diego‐Nieto, Maria B. Vidriales, Norberto Alonso‐Orcajo, Jose C. Moreno‐Samos, Francisco Martin‐Herrero, Raul Carbonell, Belen Cid, Ignacio Cruz‐Gonzalez, Javier C. Martin‐Moreiras, Carlos Cuellas, Cristina Pascual, Maria Lopez‐Benito, Pedro L. Sanchez, Felipe Fernandez‐Vazquez, Armando Perez de Prado

**Affiliations:** ^1^ Department of Cardiology University Hospital of Salamanca; ^2^ Department of Haematology University Hospital of Salamanca; ^3^ Department of Cardiology University Hospital of León; ^4^ Department of Cardiology City of Universitu Hospital of Santiago de Compostela

**Keywords:** circulating endothelial cells, endothelial progenitor cells, ticagrelor, Endothelium/Vascular Type/Nitric Oxide

## Abstract

**Background:**

Ticagrelor use during acute coronary syndromes demonstrated a decrease in all‐cause mortality in the PLATO (Platelet Inhibition and Patient Outcomes) trial. This effect has been attributed to a non–platelet‐derived improvement in endothelial function. The aim of this study was to determine differences in the number of endothelial progenitor cells and/or circulating endothelial cells found in peripheral blood in patients treated with either ticagrelor or clopidogrel during non–ST‐segment–elevation myocardial infarction.

**Methods and Results:**

In this multicenter, randomized study (NCT02244710), patients were considered for inclusion after non–ST‐segment–elevation myocardial infarction whenever they were P2Y_12_‐inhibitor naïve. Ticagrelor and clopidogrel were allocated at a 1:1 ratio. Blood samples for determining endothelial progenitor cells and circulating endothelial cells were extracted before the antiplatelet loading dose, 48 hours after presentation of index symptoms, and 1 month after the event. A multichannel cytometer was used for optimal cell characterization. A total of 96 patients fulfilled the inclusion criteria. Circulating endothelial cell levels corrected by white blood cells were as follows at baseline, 48 hours, and 1 month: 44 (28–64), 50 (33–63), and 38 (23–62) cells/mL, respectively, for clopidogrel and 38 (29–60), 45 (32–85), and 35 (24–71) cells/mL, respectively, for ticagrelor (*P*=0.6). Endothelial progenitor cell levels were 29 (15–47), 27 (15–33), and 18 (10–25) cells/mL, respectively, for clopidogrel and 20 (11–33), 22 (12–32), and 18 (11–29) cells/mL, respectively, for ticagrelor (*P*=0.9). No differences in intraindividual changes were found.

**Conclusions:**

Patients treated with ticagrelor during non–ST‐segment–elevation myocardial infarction, in comparison to clopidogrel, showed similar levels of endothelial progenitor cells and circulating endothelial cells. These data suggest that the endothelial protective effect mediated by ticagrelor is not related to bone marrow physiology modulation.

**Clinical Trial Registration:**

URL: https://www.clinicaltrials.gov. Unique identifier: NCT02244710.

**Clinical Perspective**

**What Is New?**

- Although previous studies suggested the theory that ticagrelor treatment may modulate the physiology of the endothelium in patients with an acute coronary syndrome, in this study, which was specially designed to identify variations in the number of endothelial mature and progenitor cells, we did not find any differences between clopidogrel‐ and ticagrelor‐treated patients.

**What Are the Clinical Implications?**

- Because the antiplatelet power and clinical benefits of ticagrelor have been consistently proven, the previous findings do not change the pharmacological management of acute coronary syndromes but lead to the need for more research to better understand the non–platelet‐related effects of this relatively new drug.

## Introduction

In the PLATO (Platelet Inhibition and Patient Outcomes) trial, ticagrelor showed reduced overall mortality among patients treated for acute coronary syndromes (ACSs) compared with that seen with clopidogrel^1^. The mechanism underlying this benefit has not yet been properly explained. Beyond a well‐demonstrated stronger platelet inhibition power,^2^ particularly among patients with previously higher platelet reactivity,^3^ several experimental studies have suggested that ticagrelor might exert a protective effect on the vascular endothelium.^4^, ^5^ Several small‐scale clinical trials in patients with coronary disease have reported variable results,^6^, ^7^, ^8^ so it is still unclear whether ticagrelor may exert an in vivo clinically appreciable effect; consequently, the search for an adequate explanation of this benefit has led to bigger clinical trials designed to explore the relationship between ticagrelor and cardioprotection.^9^, ^10^

The biological basis for the so‐called pleiotropic effect of ticagrelor is the increased adenosine plasma concentration due to the inhibition of erythrocyte recaptation caused by direct blocking of the ENT1 (Equilibrative nucleoside transporter 1) (solute carrier family 29 [equilibrative nucleoside transporter], member 1) receptor.^11^ Adenosine is a nucleotide with multiple implications in cellular signal transduction and energy transfer. In the vascular niche, it produces vasodilatation and promotes the normal homeostasis of arterial endothelium.^12^ Moreover, a recent study points toward adenosine as the stimulus for the migration and proliferation of endothelial progenitors, acting through its junction to the A(2B) receptor and the amplification of the *CXCR4* (C‐X‐C motif chemokine receptor 4) gene.^13^ By increasing the extracellular adenosine concentration, ticagrelor could increase the number of endothelial progenitor cells (EPCs) and improve their functionality.

Circulating endothelial cells (CECs) can be identified by their morphological characteristics and the expression of specific cell markers: von Willebrand factor and CD146. Increased CEC levels have been related to long‐term prognosis after an ACS,^14^, ^15^ and they are also considered a diagnostic criterion for defining ACSs.^16^, ^17^ In the trials cited, CEC liberation to peripheral blood showed a characteristic bimodal curve, with an early peak within the first 24 hours and a tendency for levels to rebound at 48 hours. In contrast, EPCs derive from the bone marrow and have the potential to repair and regenerate damaged endothelium after an ACS. In several clinical trials, higher EPC levels have been related to better outcome.^18^, ^19^, ^20^, ^21^

The relationship between antiplatelet drugs and endothelial cells is complex and is not well understood. Aspirin administration seems to reduce EPC levels in a dose‐dependent manner^22^ but, at the same time, improves their functionality and resistance to external aggression,^23^ so the final net effect may vary.

Considering this information, it seems crucial that clinical trials be designed to improve our understanding of the way ticagrelor and other antiplatelet drugs may modulate endothelial function, so as to identify new therapeutic targets for patients with coronary disease. The objective of this study was to find differences between EPC and CEC levels in a population of patients with non–ST‐segment–elevation myocardial infarction (NSTEMI) treated with either clopidogrel or ticagrelor.

## Methods

The data, analytic methods, and study materials will be available from the corresponding author to other researchers, on reasonable request, for purposes of reproducing the results or replicating the procedure.

### Study Population

We designed a single‐blind, multicenter, active‐treatment–controlled, randomized clinical trial. During the recruitment period, all patients admitted for NSTEMI and planned for early invasive strategy were considered for inclusion if they were P2Y_12_‐antagonist naïve (for the preceding 4 weeks). Exclusion criteria were an allergy or contraindication to aspirin, clopidogrel, or ticagrelor; high risk of bleeding or previous severe hemorrhage; active treatment with oral anticoagulants, thienopyridines, or ticagrelor; serious concomitant disease with probable fatal outcome; planned elective surgery; high chance of not completing the intended follow‐up; or pregnancy. Patients with no significant coronary artery disease and no need for revascularization were also excluded.

The final protocol was approved by the local ethics committee of the 3 centers involved, and every participant received and signed written informed consent.

### P2Y_12_‐Antagonist Administration

On admission, patients were randomized with numbered sealed envelopes and received either clopidogrel or ticagrelor at a ratio of 1:1. Other drugs administration remained at responsible physician's consideration.

### Analysis of Circulating Cells

Blood samples for flow cytometry were extracted immediately after randomization, 48 hours after the chest pain index episode, and at 1 month. They were taken by direct venipuncture, and the first 3 mL of blood was discarded to avoid contaminating the sample with local endothelial cells.

All samples were kept in preservation media (Transfix; Cytomark) to enable adequate shipping to the central laboratory. The transfer was carried out by a company specializing in biological samples management; the samples were placed in refrigerated storage, without freezing, and analyzed in a central laboratory (Hospital Universitario de Salamanca) within 5 days of extraction. The laboratory personnel were blind to the treatment allocation.

The analysis was performed with an 8‐channel flow cytometer (Becton Dickinson FACSCanto II) that allowed simultaneous multitracing of a single cellular sample. For optimal cell characterization markers, we chose CD34, CD133, CD45, CD146, CD31, KDR (CD309), 7AAD, and Syto‐16. Generic 7AAD and Syto‐16 markers were used to define cell viability and nuclear cells, respectively, to avoid contamination by debris and cell waste. The remainder of the markers were used to define specific cell populations with the following scheme:

1. CD34+, CD133−, CD45−, CD146+, and CD31+ for CECs.
2. CD34+, CD133+, CD45−, CD146+, and CD31+ for EPCs.
3. CD34+, CD133+, CD146−, and CD45^weak^ for stem hematopoietic progenitors.
4. CD34+, CD133−, CD45^weak^, and CD146− for mature hematopoietic lineages.

To determine every possible modulation that the treatment option might induce in the mentioned populations, not only was the absolute event count considered but also normalized values corrected for total white cell count were used. Intraindividual variation was studied among the 3 different time points for both the absolute event count and the normalized ratio, expressed as the difference in absolute event counts between 2 time points related to the baseline absolute event count.

### Platelet Function Assay

In association with cell population analysis, a platelet function assay was performed to detect potential relationships between platelet reactivity and CEC or EPC levels. To this end, blood samples were extracted and collected in tubes with 3.2% citrate and analyzed with the point‐of‐care analyzer VerifyNow‐P2Y_12_ (Accriva Diagnostics). The time points selected for the platelet function assays were immediately before coronary angiography, given the relationship between this time‐point assessment and the long‐term follow‐up,^24^, ^25^ and at 1 month.

### Statistical Analysis

Discrete variables were presented as absolute numbers and percentages, and continuous variables were given as mean±SD or median (interquartile range), depending on their distribution. Both χ^2^ and Fisher exact tests were used for discrete comparisons. Student *t* test and ANOVAs were selected to determine differences between continuous variables (in the case of normal distribution), and the Wilcoxon rank sum test was used for nonnormal distributions, as determined by the Shapiro‐Wilks test. The analysis of variables with repeated samples through time (CEC and EPC counts and platelet function parameters) was performed using repeated‐measures ANOVA to account for the correlations between time points.

Statistical computations were performed using the JMP 9.0.1 software from the SAS Institute.

## Results

**Figure 1:**
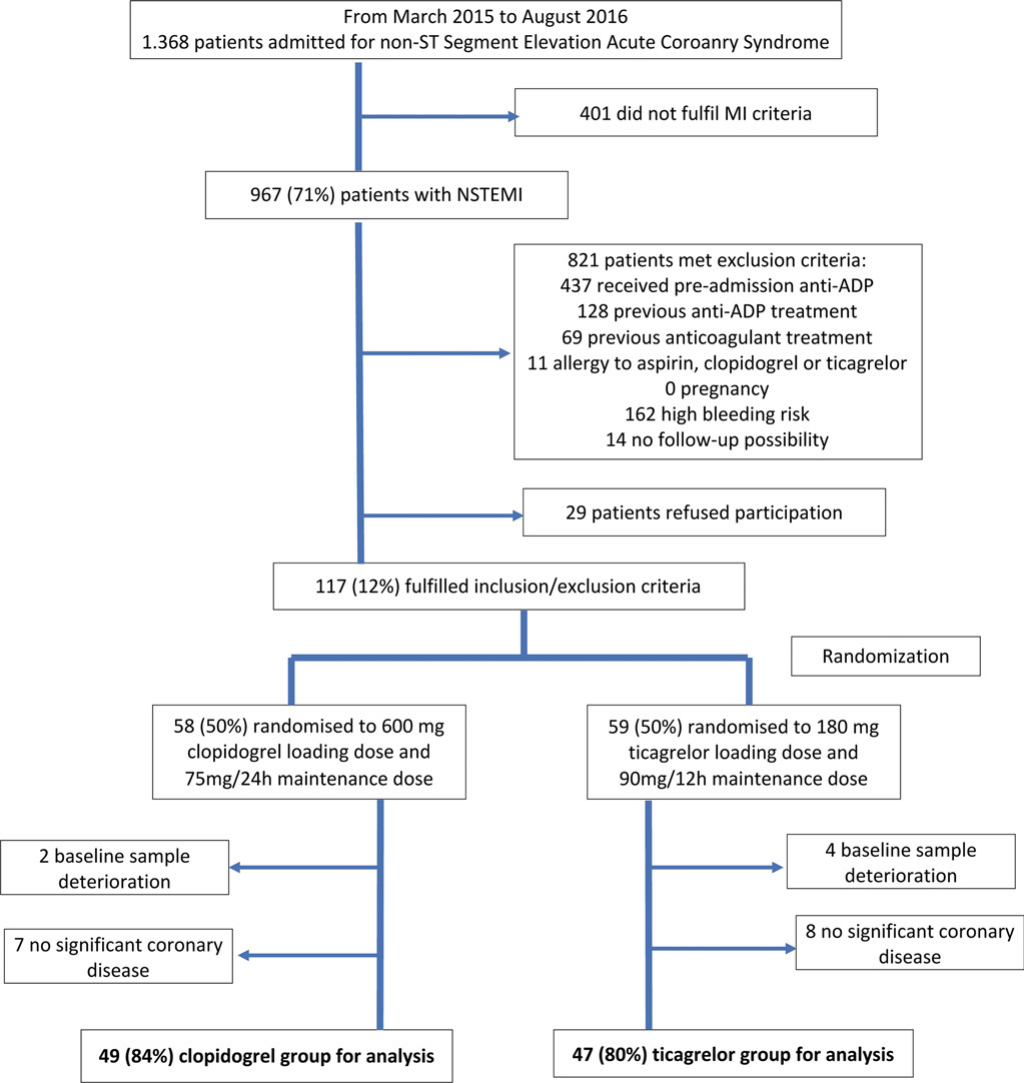
Study flow diagram. MI indicates myocardial infarction; NSTEMI, non–ST‐segment–elevation myocardial infarction.

**Figure 2:**
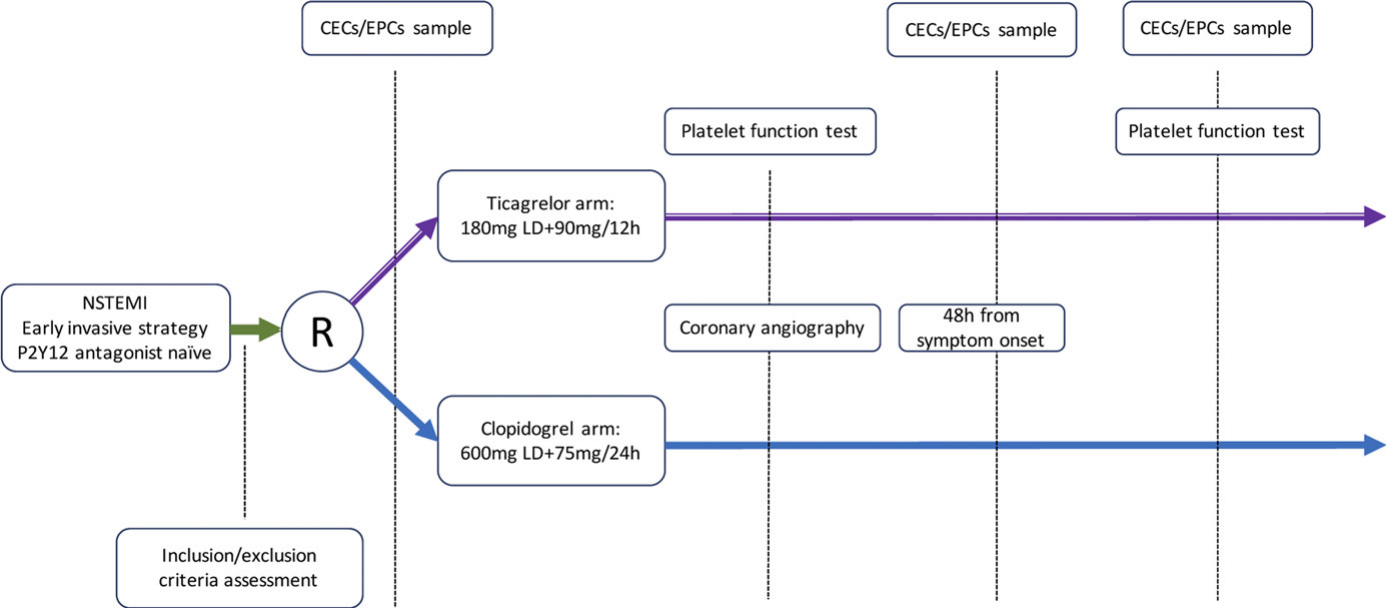
Randomization (R) and laboratory test scheme. CECs indicates circulating endothelial cells; EPCs, endothelial progenitor cells; LD, loading dose; NSTEMI, non–ST‐segment–elevation myocardial infarction.

Between March 2015 and August 2016, 117 patients were randomized. After 15 were excluded for the absence of coronary artery disease and 6 for baseline sample deterioration, 96 were finally selected for analysis. The study flow diagram and laboratory test scheme are shown in Figures 1 and 2.

### Baseline Characteristics

The baseline characteristics of the patients are summarized in Table 1. No significant differences were found between the 2 treatment groups.

**Table 1:** Baseline Characteristics

	Clopidogrel (n=49)	Ticagrelor (n=47)	*P* Value
Age, y	67.7	65.6	0.4
Male	37 (77)	39 (85)	0.3
Body mass index	27.8±3.4	28.3±3.7	0.5
Diabetes mellitus	7 (14.6)	11 (23.9)	0.3
Arterial hypertension	26 (54.2)	23 (50)	0.7
Dyslipidemia	22 (45.8)	29 (63)	0.1
Smoker	13 (27)	9 (19.6)	0.4
Cardiac history			
Previous MI	3 (6.4)	4 (8.7)	0.7
Previous PCI	3 (6.4)	4 (8.7)	0.7
Previous CABG	1 (2.1)	3 (3.2)	0.5
Previous stroke	0 (0)	1 (2.1)	0.3
Drugs administered			
Aspirin	49 (100)	46 (100)	0.9
Statins (atorvastatin 80 mg/24 h, n=96)	40 (93)	38 (92.7)	0.9
β‐Blockers	27 (62.8)	30 (75)	0.2
ACEIs (ramipril, n=85; captopril, n=11; dosage depending on arterial tension)	32 (74.4)	26 (66.7)	0.4
Nitrates	22 (52.4)	25 (62.5)	0.4
Angiography and intervention			
Coronary disease			
None (0 vessels)	5 (11.4)	8 (19.5)	0.6
Single‐vessel	19 (43.1)	15 (36.6)	
Multivessel	20 (45.5)	18 (43.9)	
Left main disease	1 (2.1)	4 (9.1)	0.14
Graft disease	1 (2.1)	1 (2.3)	0.9
Radial access	44 (93.4)	41 (93.2)	0.9
No stent	1.4±1.1	1.4±1.5	0.9
Laboratory values			
Hemoglobin, mg/dL	14.9±1.7	14.7±1.6	0.5
Creatinine, mg/dL	0.9±0.3	0.9±0.3	0.4
Glycemia, mg/dL	109 (91–132)	114 (100–139)	0.7
CK maximum, UI/L	245 (141–426)	279 (131–431)	0.4
Platelets, ×1000/mL	203 (178–241)	198 (171–238)	0.9

Data are shown as mean±SD, median (interquartile range), or n (%) except as noted. ACEIs indicates angiotensin‐converting enzyme inhibitors; CABG, coronary artery bypass grafting; CK, creatine kinase; MI, myocardial infarction; PCI, percutaneous coronary intervention.

### Platelet Function

**Figure 3:**
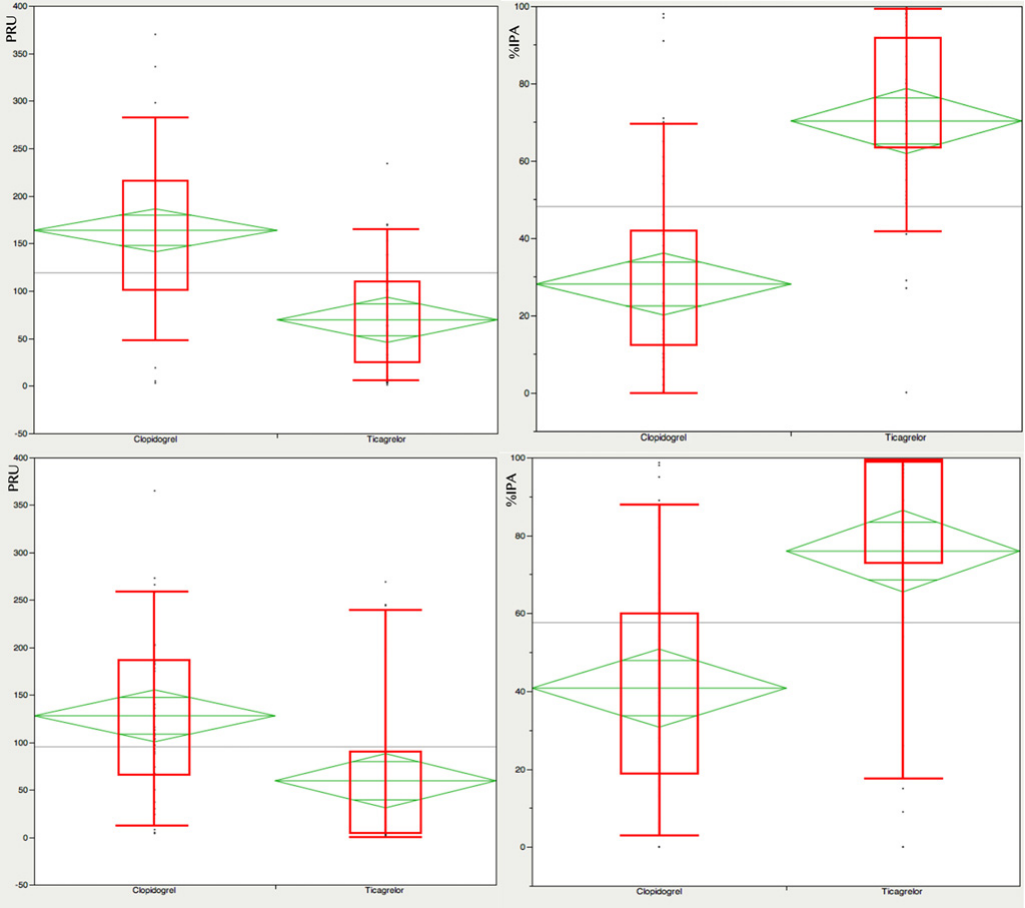
Platelet function determination related to treatment option allocation and mean comparison with the Student *t* test and ANOVA. Upper panels: analysis during coronary angiography. Lower panels: analysis performed 1 month after procedure. %IPA indicates percentage of platelet inhibition; PRU, platelet reaction units.

Patients treated with ticagrelor showed greater platelet inhibition, as determined by the VerifyNow‐P2Y_12_ system. The values are expressed in platelet reaction units and percentage of inhibition of platelet aggregation (determined with respect to ADP‐independent reactivity). The VerifyNow data are summarized in Table 2 and Figure 3.

**Table 2:** Platelet Reactivity During Coronary Angiography and 1 Month After the Procedure

	Clopidogrel	Ticagrelor	*P* Value
PRU at angiography	176 (97–210)	60 (24–108)	<0.0001
IPA% at angiography	17 (5–48)	73 (57–89)	<0.0001
PRU at 1 mo	111 (77–182)	21 (4–80)	0.001
IPA% at 1 mo	37 (17–62)	89 (71–98)	<0.0001

Data expressed in the median (interquartile range). IPA indicates inhibition of platelet aggregation; PRU, platelet reaction units.

### Flow Cytometry

**Figure 4:**
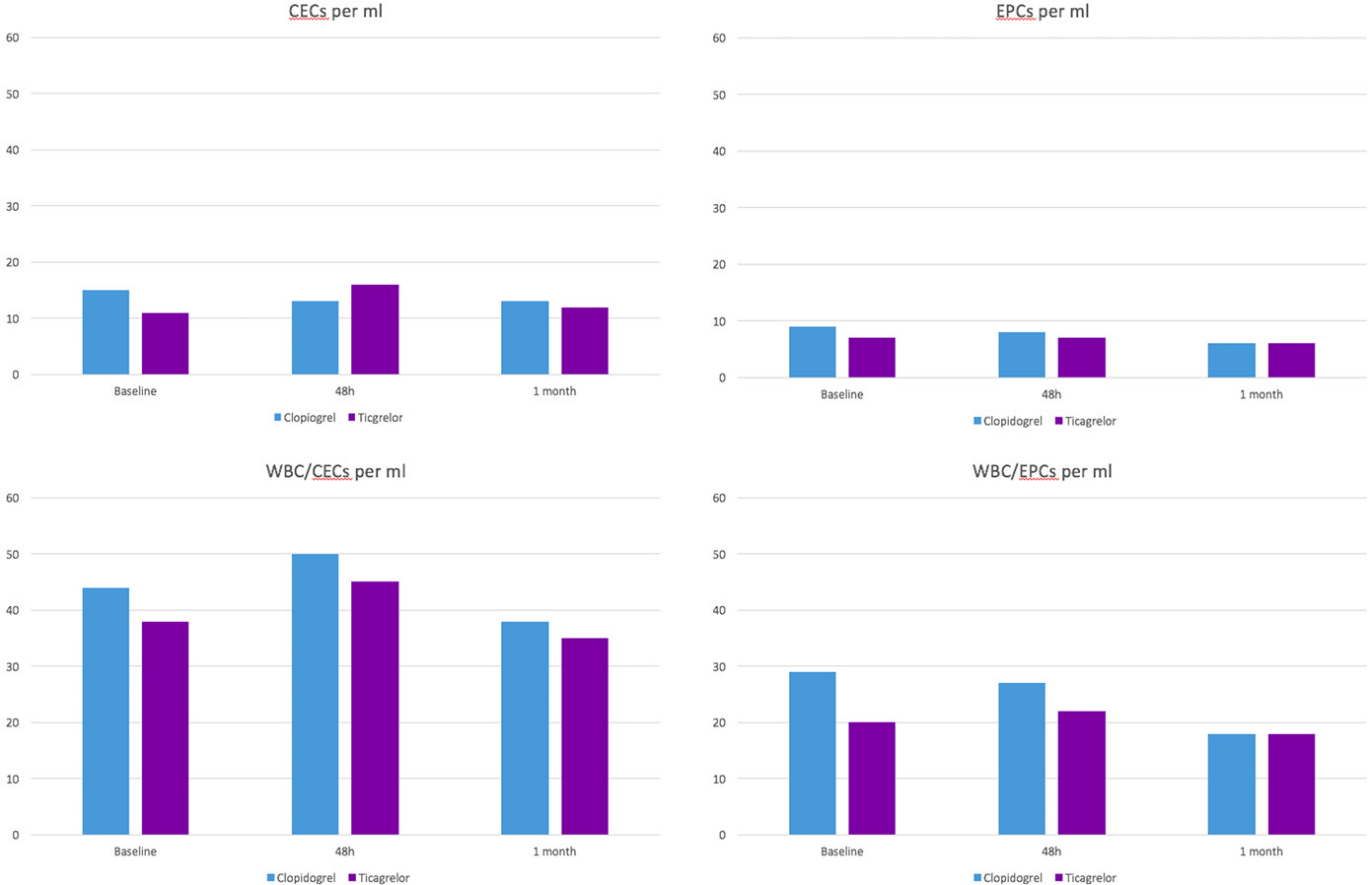
Flow cytometry. Event count per milliliter expressed as the median (interquartile range). CECs indicates circulating endothelial cells; EPCs: endothelial progenitor cells; WBC/CECs, ratio of white blood cells/circulating endothelial cells (normalized events); WBC/EPCs, ratio of white blood cells/endothelial progenitor cells (normalized events).

We looked for differences between populations, both in absolute event counts in the flow cytometer and corrected for the total number of white cells in the sample (normalized, ratio of white blood cells/CECs, and ratio of white blood cells/EPCs). Intraindividual variation among the 3 different time points was also determined. We identified a slight increment in the overall white blood cell/CEC levels at 48 hours, as described previously, from baseline levels of 41 cells (interquartile range: 28–60) to 48 cells (interquartile range: 33–77; *P*=0.03); however, no difference was detected between the 2 treatment groups at any time. Flow cytometry data are summarized in Figure 4 and Tables 3, 4 through 5.

**Table 3:** Flow Cytometry of CECs and EPCs, Total Event Count Per Milliliter

	Clopidogrel	Ticagrelor	*P* Value
CEC baseline	15 (8–22)	11 (9–18)	0.8
CEC 48 h	13 (9–20)	16 (11–26)	0.8
CEC 1 mo	13 (7–20)	12 (9–21)	0.8
EPC baseline	9 (6–15)	7 (4–10)	0.08
EPC 48 h	8 (5–11)	7 (4–10)	0.4
EPC 1 mo	6 (4–8)	6 (4–9)	0.6

Data shown as median (interquartile range). CEC indicates circulating endothelial cells; EPC, endothelial progenitor cells.

**Table 4:** Flow Cytometry of CEC and EPCs, Normalized Count Corrected by White Cell Count in the Sample

	Clopidogrel	Ticagrelor	*P* Value
WBC/CEC baseline	44 (28–64)	38 (29–60)	0.6
WBC/CEC 48 h	50 (33–63)	45 (32–85)	0.7
WBC/CEC 1 mo	38 (23–62)	35 (24–71)	0.6
WBC/EPC baseline	29 (15–47)	20 (11–33)	0.6
WBC/EPC 48 h	27 (15–33)	22 (12–32)	0.4
WBC/EPC 1 mo	18 (10–25)	18 (11–29)	0.9

WBC/CEC indicates ratio of white blood cells/circulating endothelial cells (normalized count); WBC/EPC, ratio of white blood cells/endothelial progenitor cells (normalized count).

**Table 5:** Flow Cytometry, Intraindividual Variation of CECs and EPCs Expressed Both in Total Event Count and Normalized Count by White Cells in the Sample

	Clopidogrel	Ticagrelor	*P* Value
Total CEC variation			
Absolute count			
Baseline vs 48 h	0 (−6 to 7)	3 (−2 to 14)	0.9
Baseline vs 1 mo	0 (−9 to 10)	3 (−7 to 7)	0.9
% variation			
Baseline vs 48 h	3 (−71 to 47)	27 (−18 to 54)	0.4
Baseline vs 1 mo	−2 (−118 to 50)	25 (−83 to 58)	0.4
Total EPC variation			
Absolute count			
Baseline vs 48 h	0 (−6 to 2)	0 (−3 to 3)	0.3
Baseline vs 1 mo	−2 (−7 to 3)	0 (−4 to 2)	0.3
% variation			
Baseline vs 48 h	−4 (−117 to 361)	0 (−50 to 41)	0.3
Baseline vs 1 mo	−47 (−122 to 36)	0 (−80 to 28)	0.7
WBC/CEC variation			
Absolute count			
Baseline vs 48 h	6 (−16 to 22)	13 (−10 to 47)	0.9
Baseline vs 1 mo	1 (−23 to 32)	7 (−22 to 27)	0.6
% variation			
Baseline vs 48 h	13 (−28 to 47)	28 (−36 to 58)	0.7
Baseline vs 1 mo	4 (−179 to 50)	20 (−84 to 39)	0.3
WBC/EPC variation			
Absolute count			
Baseline vs 48 h	−2 (−20 to 10)	−0.1 (−11 to 9)	0.7
Baseline vs 1 mo	−5 (−19 to 5)	−5 (−12 to 3)	0.7
% variation			
Baseline vs 48 h	−11 (−108 to 44)	−0.2 (−71 to 47)	0.3
Baseline vs 1 mo	−33 (−127 to 17)	−23 (−91 to 12)	0.7

CEC indicates circulating endothelial cell; EPC, endothelial progenitor cell; WBC/CEC, ratio of white blood cells/circulating endothelial cells (normalized count); WBC/EPC, ratio of white blood cells/endothelial progenitor cells (normalized count).

### Statistical Statement

Based on the results of previous studies of CECs^9^, ^16^ and EPCs,^20^, ^26^ the initial plan was to recruit at least 80 patients to identify differences of 6.34 with a SD ±10 cells to obtain 80% statistical power with a risk of α=0.05. However, the post hoc results were very different from what was predicted, especially with a much larger standard deviation than expected. Consequently, the actual statistical power to detect the previously measured difference of 6.34 was smaller (54%). With the observed SD ±25 normalized cells, the sample size required to detect a difference of 6.34 with 80% statistical power and risk of α=0.05 is 178.

## Discussion

The results of this study show no differences, at any time point, in the number of CECs and EPCs between patients treated with clopidogrel and those treated with ticagrelor. These results contradict the hypothesis of potential modulation of the endothelial function through changes in the number of endothelial cells induced by ticagrelor, as well as the results recently reported by Bonello et al.^26^ Nevertheless, we must emphasize the difficulty of endothelial cell identification, given the number of cell markers needed to properly characterize them and because this population represents a negligible fraction of the total white nuclear cells in peripheral blood. For this reason, precisely determining such minimal levels is, at the very least, intimidatingly difficult. The study carried out by Bonello et al^26^ involved cell characterization with a 3‐channel cytometer, meaning they needed to process the sample several times and change markers, with a consequent loss of precision. In addition to this procedural issue, the unique cell subpopulation that showed differences between treatment options was defined as CD34+ and KDR+; various concerns about the limitations of this identification method have been reported previously.^27^

The greatest strength of our study is the use of a high‐end, latest generation, 8‐channel cytometer and a laboratory team with enormous experience in the cell characterization necessary for oncohematology, for which reason we believe the measured levels are accurate. Nevertheless, every trial aimed at determining CECs and/or EPCs highlights the small quantities of these cells that can be identified among the circulating white cells. With an absolute count barely exceeding a couple of tens of cells in the best scenario and such a wide distribution of values, it is logical to deduce that minimal variations are not easy to assess. In addition, several methods of EPC characterization have been reported with variable results,^28^ so the accuracy of the antigenic profile used to identify CECs and EPCs has, to date, been questionable. A basic bench science reevaluation of the methods has been suggested before we can transfer them to clinical trials.^29^

Independent of that study limitation, it seems likely that the protective effect that adenosine may exert over the vascular endothelium is mediated in other, more complex ways than simply increased cell number. Moreover, it is possible that the extracellular level of adenosine that ticagrelor induces through blocking the ENT1 receptor is clinically irrelevant. In the AMISTAD‐II (Acute Myocardial Infarction Study of Adenosine‐II) trial,^30^ reduced myocardial scarring after continuous infusion of adenosine was obtained with a dose of 70 μg/kg per minute, and no effect was demonstrated with lower doses or a placebo.

It is true that the increase in adenosine level that ticagrelor can promote is sustained over time, and this may explain the benefit reported in an experimental swine model of infarction and reperfusion.^31^ Nevertheless, the findings shown in that particular study must be read with caution, given the limitations of experimental studies performed on young healthy animals.

Other recent small clinical trials that aimed to better understand the relationship between ticagrelor and endothelium showed variable results. Campo et al^32^ recently reported a protective effect of ticagrelor compared with clopidogrel, consisting of improvements in surrogate endothelial function parameters (rate of apoptosis and nitric oxide levels in human umbilical vein endothelial cells and levels of reactive oxygen species in peripheral blood mononuclear cells), but found no differences in the circulating levels of 29 human cytokines after 1 month of treatment. The authors concluded that the underlying mechanism of such endothelial protection remains unclear. Even the improvements in endothelial function itself can be challenged. Another trial, recently published by Ariotti et al, using different endothelial function parameters (eg, pulse amplitude tonometry assessment) could not find differences among patients treated with ticagrelor, clopidogrel, or prasugrel in a crossover‐designed model.^8^

At this point, waiting for the results of clinical trials conducted to identify the capacity of ticagrelor to induce cardioprotection during ACS (clinical trials TAPER‐S [Ticagrelor and Preconditioning in Patients with coronary Artery disease, NCT02701140] and ETCH [A Trial Comparing the Ischemic Preconditioning Effects of Ticagrelor and Clopidogrel in Humans NCT01743937]), it seems reasonable to assume that the benefit in overall mortality obtained with ticagrelor compared with clopidogrel cannot be separated from its inherently stronger antiplatelet effect and generally improved efficacy. The PRACTICAL study, a real‐life European study performed with a nonselected population from SWEDEHEART (Swedish ACS registry), confirms the overall reduction in mortality with the use of ticagrelor compared with clopidogrel^33^ and reinforces the results of the PLATO trial; however, when compared with prasugrel—a drug with the same antiplatelet power—in another real‐life registry, it is unclear whether such differences actually exist.^34^ Because the main cause of death among these patients is the occurrence of new thrombotic events, it seems reasonable to state that the higher the platelet inhibition achieved, the less the overall likelihood of mortality.

### Study Limitations

As noted earlier, the main limitation of this study is the difficulty inherent in the process of identifying EPCs and CECs with the actual available laboratory methods. In addition, the sample size was not large enough. Although predicting an appropriate sample size based on published results suggested that a sample of 80 would be adequate, post hoc analysis using the larger observed standard deviation suggested that a larger sample (178) was needed.

The design of the study, comparing ticagrelor and clopidogrel, was a decision made to explore the findings of the PLATO trial. Using a more modern approach, comparison with an equivalently powerful anti‐ADP agent such as prasugrel could report more practical, up‐to‐date information.

## Conclusions

In our study, no differences were found between ticagrelor and clopidogrel regarding circulating levels of CECs and/or EPCs. This finding suggests that the better clinical results achieved with ticagrelor in clinical trials might be driven by other mechanisms that need to be studied in future research.

## Sources of Funding

This study has been supported by AstraZeneca (ISSBRIL0205) and the Fundación Investigación Sanitaria en León.

## Disclosures

Dr Diego‐Nieto reports speech fees from AstraZeneca and Daiichi‐Sankyo; Dr Pérez de Prado reports research grants from AstraZeneca. The remaining authors have no disclosures to report.
